# Supplementary material for: DNA-RNA Hybrid (R-Loop): From a Unified Picture of the Mammalian Telomere to the Genome-Wide Profile
Source: Cells. 2021 Jun 19;10(6):1556. doi: 10.3390/cells10061556 (PMC8233970; doi:10.3390/cells10061556)
Supplement: Supplementary file 1 [file cells-10-01556-s001.zip › cells-1220888-SI.pdf]

Supplementary Figures

DNA-RNA hybrid (R-loop):
from a unified picture of the mammalian telomere to the genome-wide profile

Minoo Rassoulzadegan[1*], Ali Sharifi-Zarchi[2] and Leila Kianmehr[3]
(1) Université Cote d'Azur (UCA) , INSERM-CNRS, IRCAN, 06107 Nice France
(2) Computer Engineering Department, Sharif University of Technology Tehran, 1458889694, Iran sharifi@sharif.edu
(3) Animal Sciences and Biotechnology Department, Faculty of Life Sciences and Biotechnology,
Shahid Beheshti University, Tehran 1983963113, Iran; kianmehr96@gmail.com
Running title: **R-loop in paternal genome**
Key words: sperm, TERRA, telomere, R-loop, genome-wide transcripts
*corresponding author: minoo@unice.fr

Supplementary Figures and Tables

Supplementary Figure S1a. Schema of the nucleic acid extraction protocol and blot analysis.

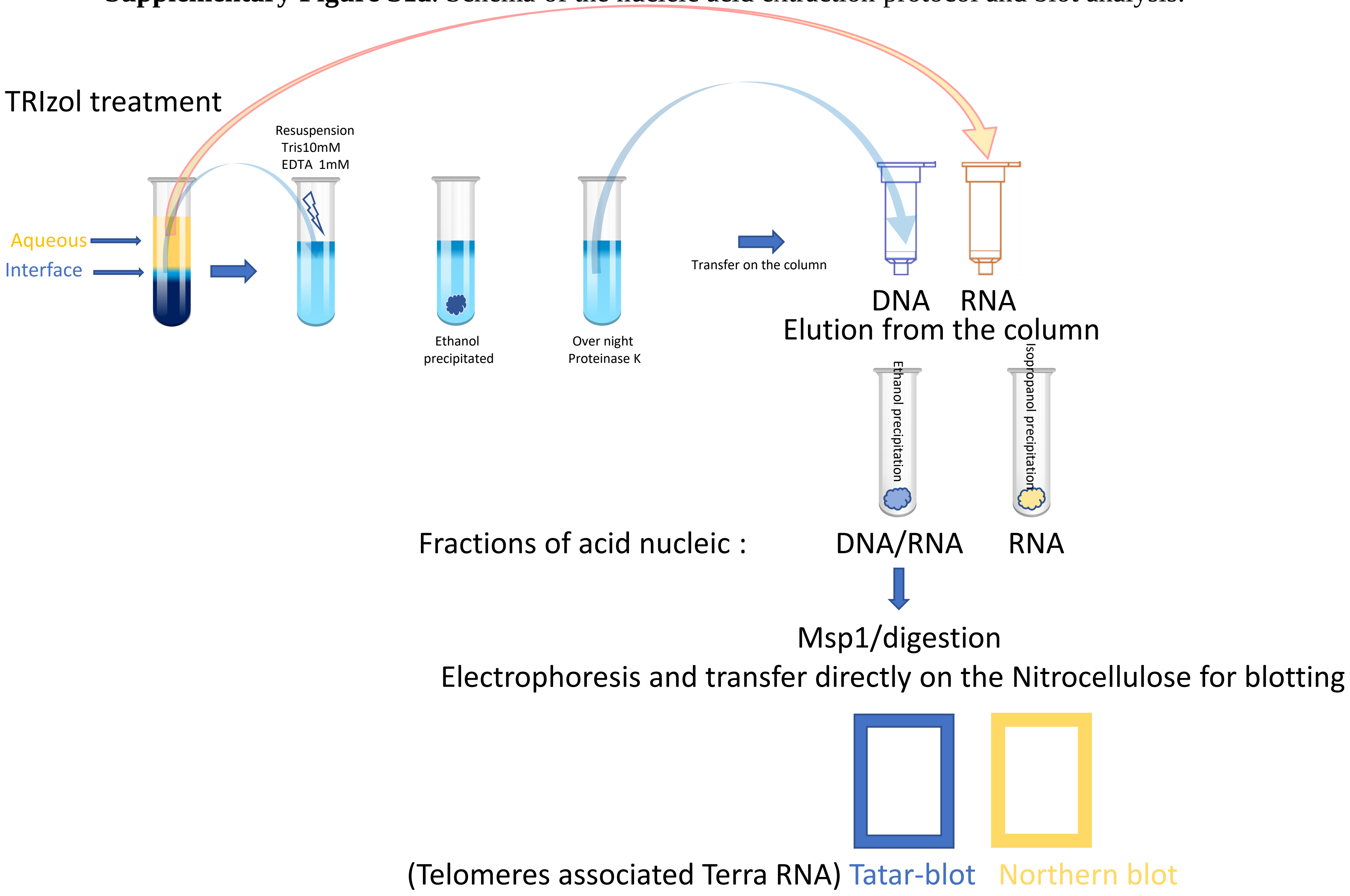

Supplementary Figure S1b. Schema of the nucleic acid extraction protocol and for samples preparation for RNA sequencing analysis.

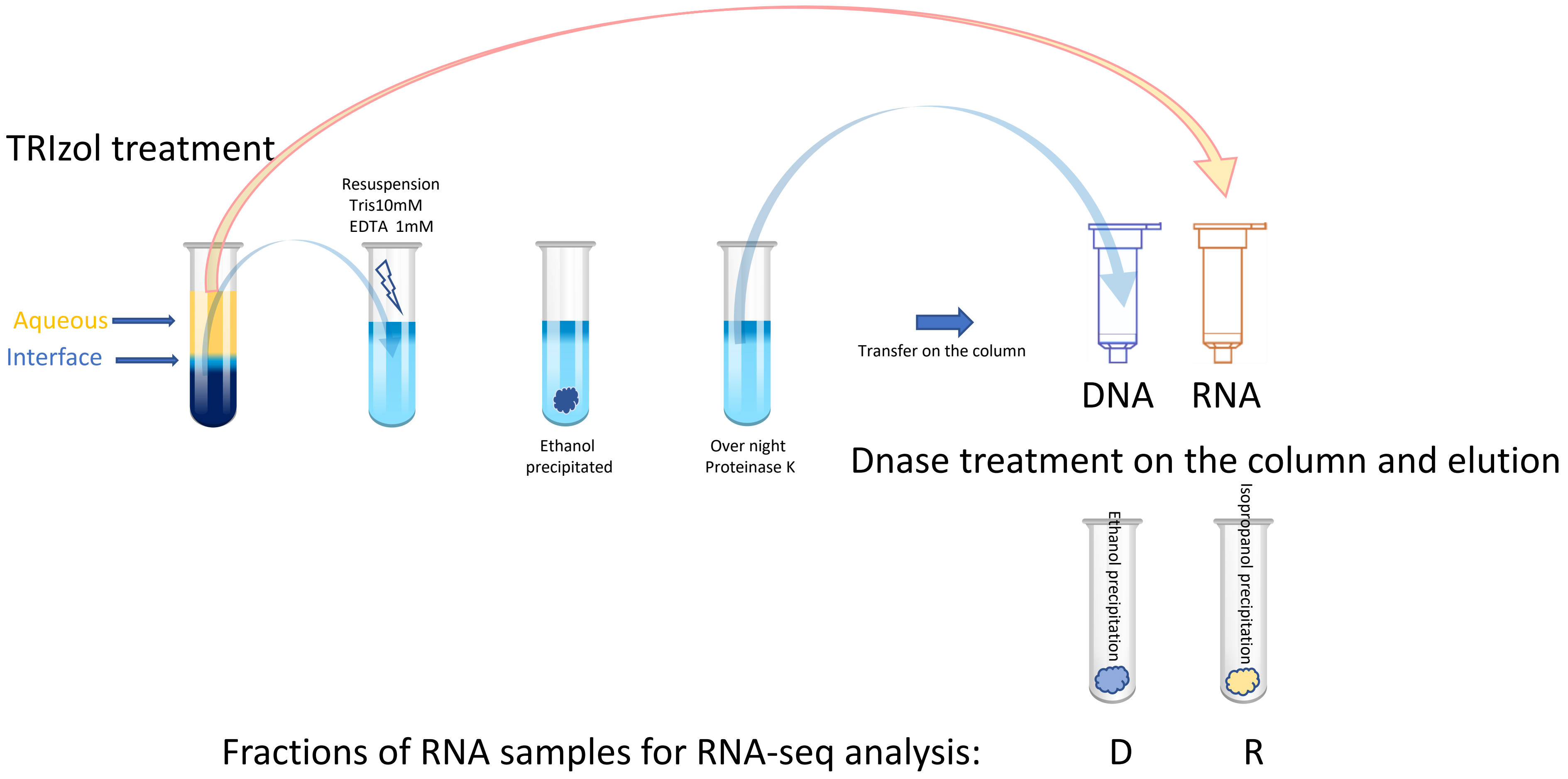

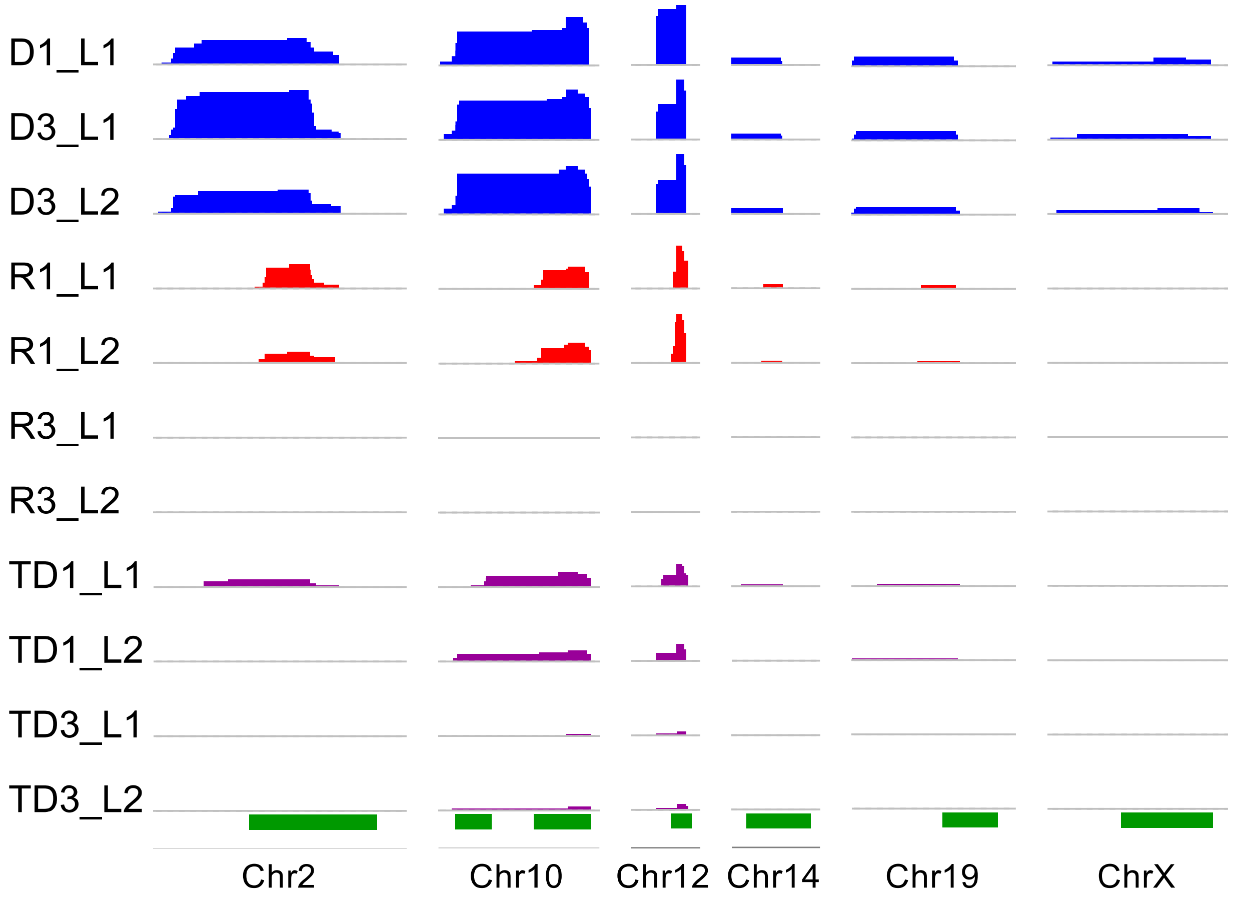

Supplementary Figure S2. RNA-seq signals over TERRA sequences at q-arm telomeric regions of several mice chromosomes. Each track shows a different sample 1 (male of six months) and 3 (male of 14 month) D1-D3 are DNA-bound RNA, R1-R3 are cytoplasmic RNA from sperm, and TD1-TD3 are DNA-bound RNA from testes. The heights of the peaks show normalized expression level (number of reads per 10^7 reads) over each genomic region, with equal 0-50 scale. The green track shows location of TERRA sequences in mice genome (minimum four consecutive TTAGGG repeats).

Supplementary Figure S3.

Part of Bioinformatics method for Supplementary Fig. 3 description:

Reads with a length of less than 20 bp were discarded. Samples were then directly quantified using Salmon v0.12.0[38] on an index built from the GRCm38 genome using GENCODE vM18. RNA composition estimation (Supplementary Fig. 3) were created by summing the reads of transcripts from the same RNA biotype and dividing by the total number of assigned reads in each sample[39].

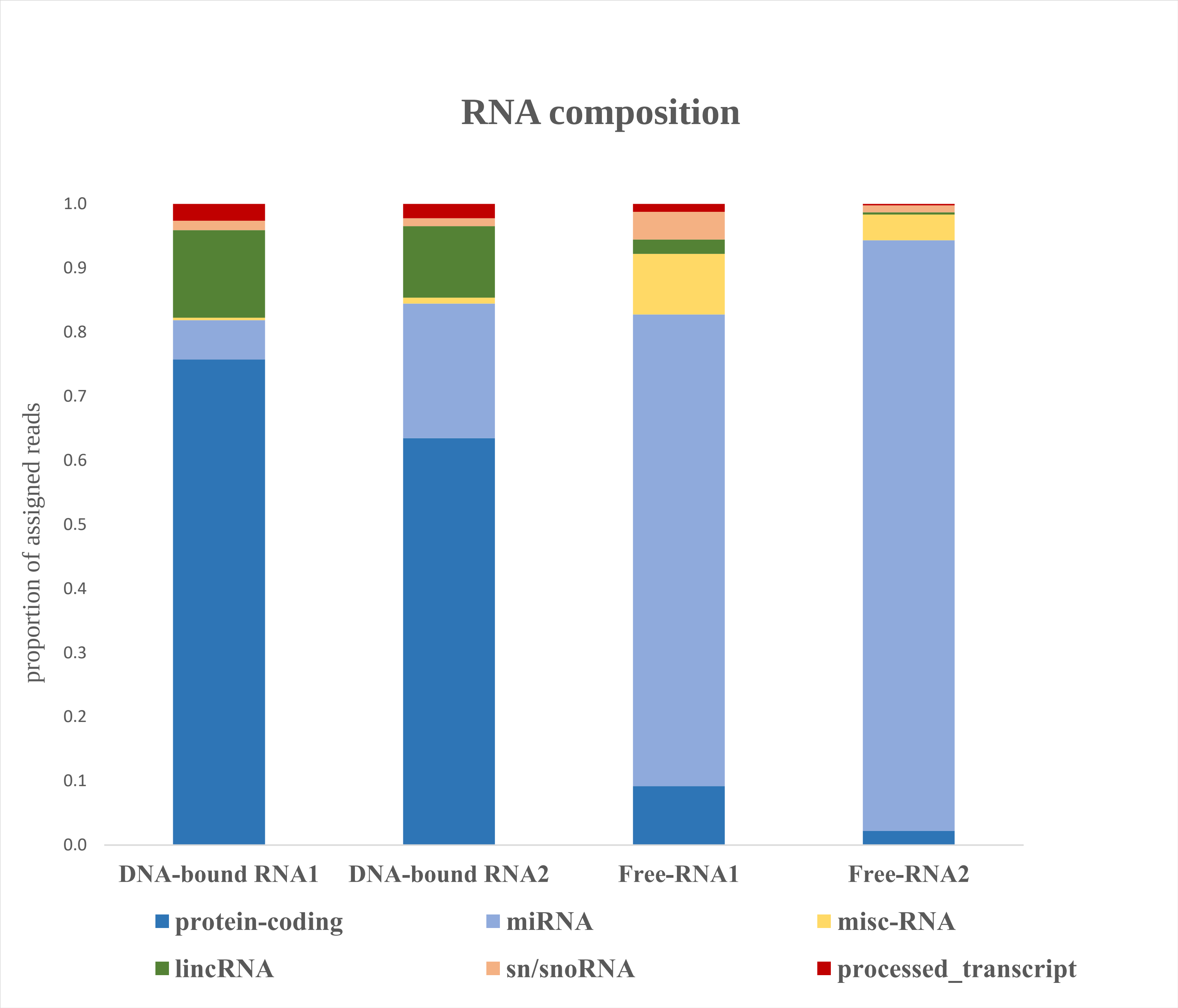

a.RNA composition revealed by high-throughput RNA sequencing in sperm. Sperm transcriptome of free-RNA and DNA-bound RNA, resulting from individual mice (6 and 14 months old) reveals variable levels of transcripts mapping to protein-coding RNA, microRNA (miRNA), miscRNA, long intergenic non-coding RNA (lincRNA), sn/snoRNA and processed-transcript*. Y-axis shows the proportion of assigned reads in biological replicates of each sample group.

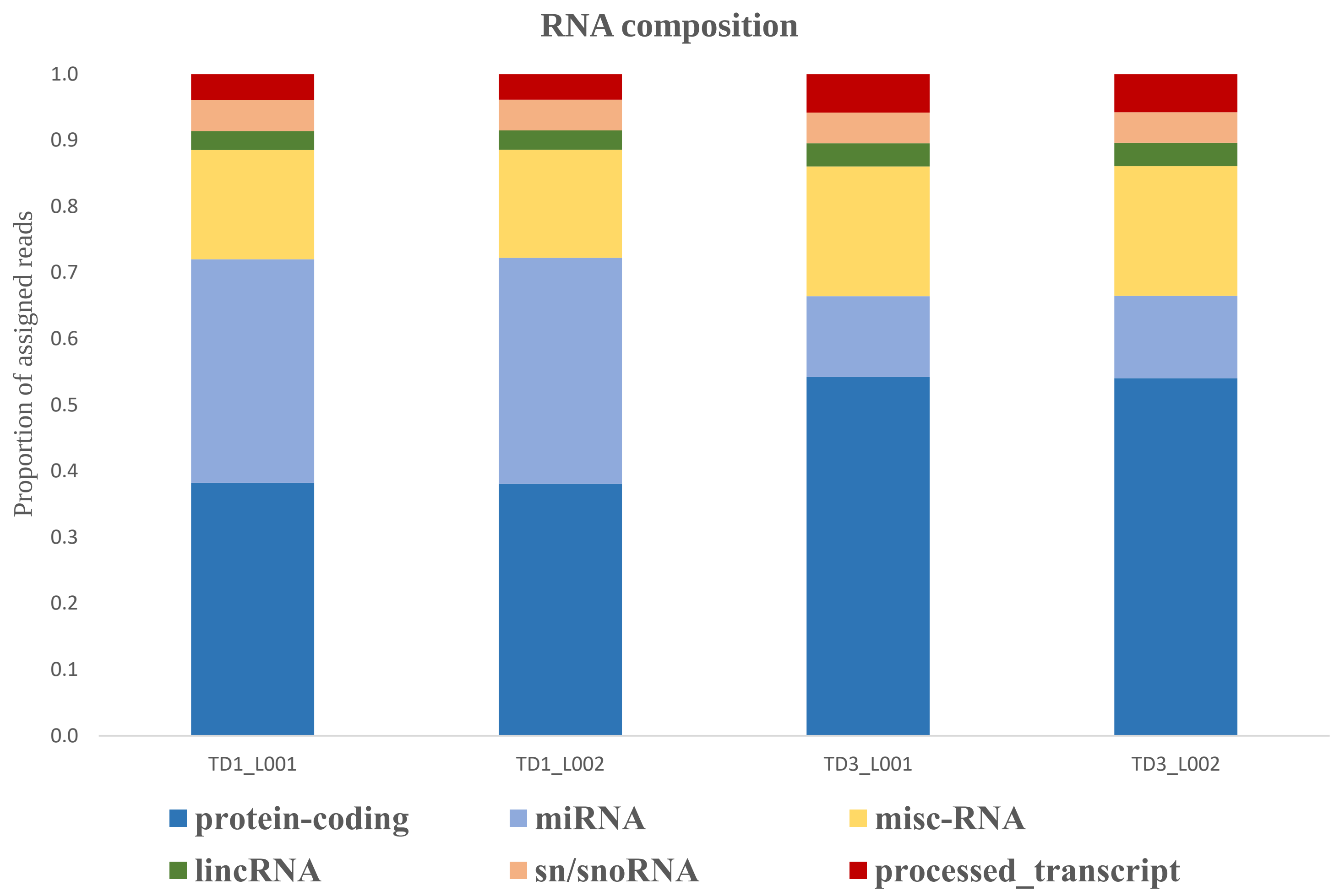

b. RNA composition revealed by high-throughput RNA sequencing in testis. Transcriptome of testis of DNA-bound RNA (TD), resulting from individual mice (6 and 14 months old) reveals levels of transcripts mapping to protein-coding RNA, microRNA (miRNA), miscRNA, long intergenic non-coding RNA (lincRNA), sn/snoRNA and processed-transcript*. Y-axis shows the proportion of assigned reads in biological replicates of each sample group.

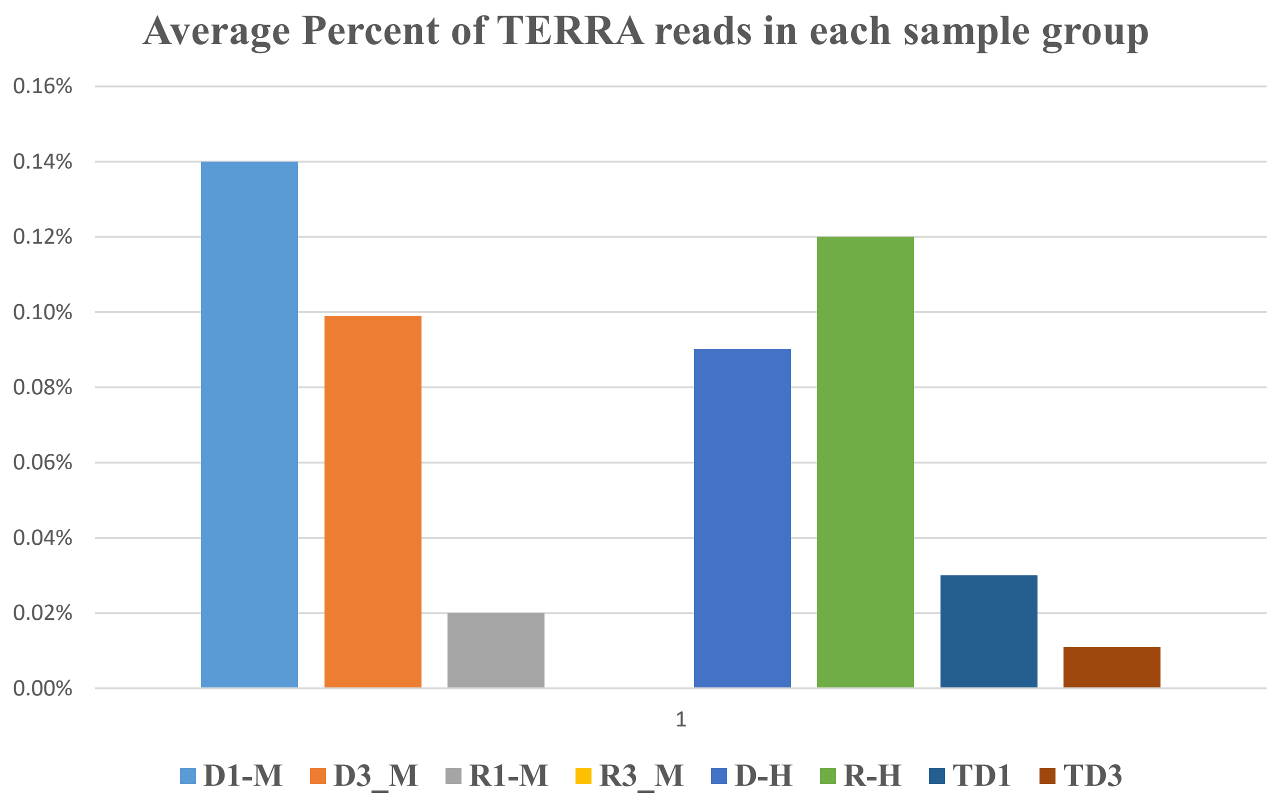

c. Percent of TERRA* (minimum four consecutive CCCTAA repeats) revealed by high-throughput RNA sequencing of sperm of mouse DNA-bound RNA (D-M) sperm, TD1, TD3 testes and human DNA-bound RNA (D-H) and free-RNA (R-H), Y-axis shows the TERRA percent of assigned reads.

Wide expression signals of transcripts over mouse chromosomes.

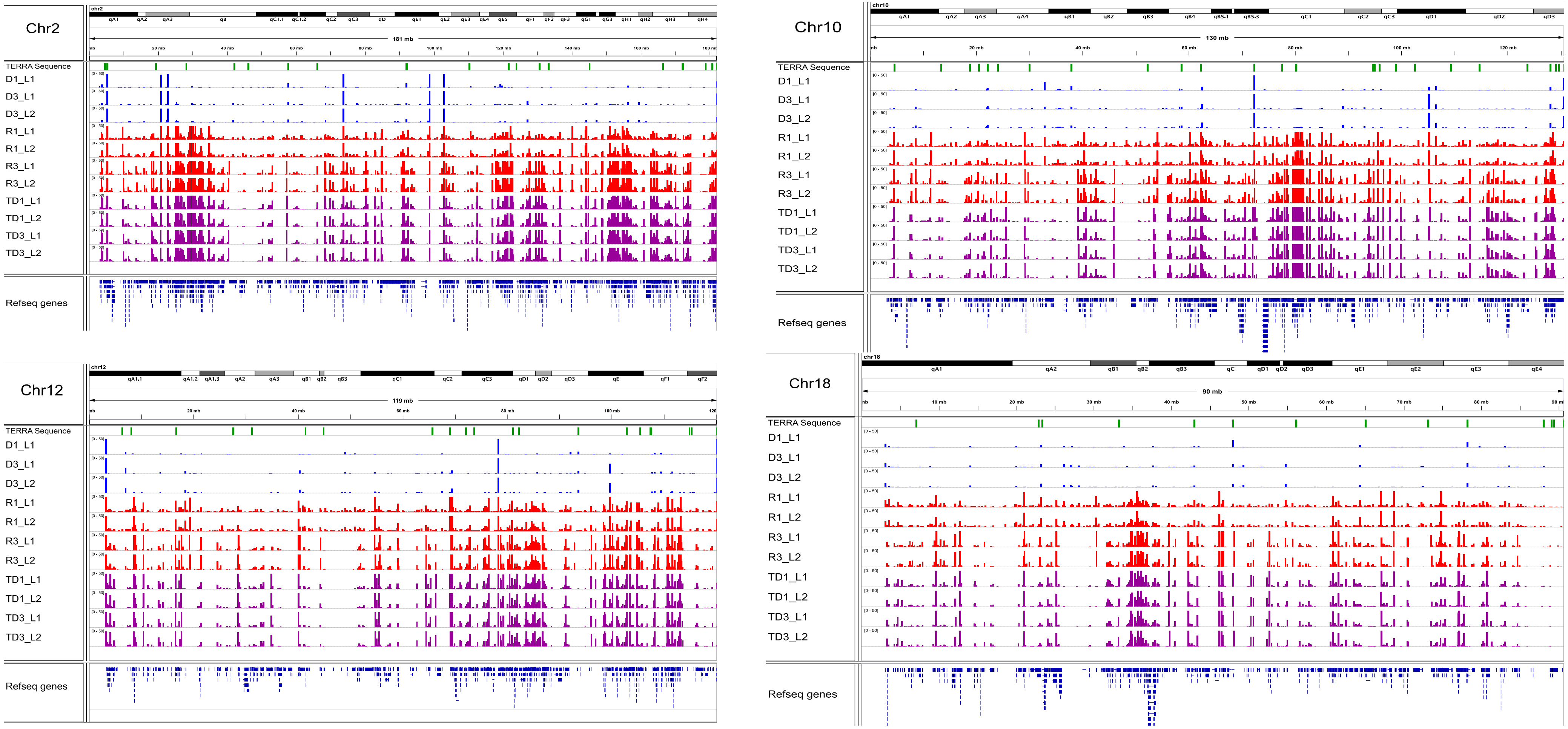

Supplementary Figure S4. Examples of landscapes expression signals of the chromosomes 2, 10, 12 and 18 (mice). For each chromosome, the green bars shows the location of TERRA-homologous regions (minimum 4 consecutive TTAGGG repeats). The following tracks show different sperm samples (D1-D3 are DNA-bound RNA, R1-R3 are cytoplasmic RNA, and TD1-TD3 are from testes DNA-bound RNAs). The heights of the peaks show normalized expression level (number of reads per 10^7 reads) over each genomic region, with equal 0-50 scale. Known genes in RefSeq database are located at the bottom of each panel.

Peak annotation on entire genome.

Supplementary Figure S5a. DRNA intergenic regions components

Detailed Annotation	number
Simple_repeat*	4212
Low_complexity	400
LINE	184
SINE	104
LTR	102
Satellite	87
rRNA	35
DNA	3
srpRNA	2
Other	2
snRNA	1

5b. Number of simple repetitive sequences in DRNA intergenic regions

Motif	N	Motif	n
GAAA	1201	TTTC	1053
GAA	551	TTC	517
GGAAA	113	TTTCC	93
GGAA	86	GA	83
TC	83	GAAAA	67
TTTTC	57	TTCC	56
TTAGGG	33	CCCTAA	24
TTCTC	24	TTCTCC	14
GGGAA	11	GGA	7

Supplementary Table S1

List of mouse and human tissues and culture cell lines

Species	Tissues ex vivo	ALT status	Restriction cleavage	TaTR signal
Mouse tissues ex vivo	Embryo fibroblasts	Negative	MspI	++
	Testis	ND	MspI	+++
	Sperm	ND	MspI	+++++
	Brain	ND	MspI	++
Mouse cell lines	15P1, PY6, 45T1,MTT3 CCL39TK	ND	MspI	+
Rat cell lines	FR3T3, FR-RAS, BPV-1, BPV-5, SVWTAII, SWTN2, WTRSV	ND	MspI	+
Human cell lines	RAJI<br>HELA<br>293<br>human U-2 OS cancer cell line (ATCC® HTB-96™)	Positive<br>ND<br>ND<br>Positive	MspI	+
Human	Saliva<br>Sperm	ND<br>ND	MspI<br>MspI	+++<br>+++

Supplementary Table S2 Deoxyribonucleotide probes

Subtelomeric sequences [20]	Probe sequence
Chromosome 18 short arm (NT_039674.8)	GGGACATCTTCTGGATATATGCCCAGGAGAGGTATTG<br>CAATACCTCTCCTGGGCATATATCCAGAAGATGTCCC
Chromosome 18 long arm (NT_039678.8)	ACAGAGGTGAGGCCAGTGTCAGTCTGTCCCTTCTTT<br>TGCCACAGATTTTAATATTAAACACATATAAAATA<br>TATTTTATATGTGTTTAATATTAAAATCTGTGGCA
Chromosome 9 short arm (NT_039471.8)	TCACGTTTTTCAGTGATTTTGTCATTTTTCAAGTTGT<br>ACAACTTGAAAAATGACAAAATCACTGAAAAACGTGA
Chromosome 9 long arm (NT_166313.2)	CTTGGCTGTCTTGGAATTCACTTTGTAGACCAGGCT<br>CTGTCTCCCTGGGATTAAAGGCATGCACCACCATGC<br>CTCTTTTATGCATTTTGAGATGATCTTGTCCTGGAAAG<br>CTTTCCAGGACAAGATCATCTCAAAATGCATAAAAGAG
Chromosome 17 long arm (NT_039649.8)	GGCAGAGGCAAGAGGATCTCTGTGAATTCCAGGTAAG<br>CTTACCTGGAATTCACAGAGATCCTCTTGCCTCTGCC
Chromosome 19 long arm (NT_082868.6)	GCTGAATAGATGGTTCTATGTTTATTCCTAAGAAATAG<br>GTTTATTGGATCTTCACAGGAATACCCCACTCTGCTG<br>GCTGCAGAGTGGGGTATTCCTGTGAAGATCCAATAAAC
Chromosome X long arm (NT_165788.2)	GTCTGGGTCTTTGGAGAGGCTGGCTTAGGGGCTAAAG<br>CTTTAGCCCCTAAGCCAGCCTCTCCAAAGACCCAGTC
Chromosome X short arm (NT_039699)	GCAGAGCATTCAGTACTCACTAACC<br>GGTTAGTGAGTACTGAATGCTCTGC
Chromosome Y (NT_187051.1)	GTTTGTACGGAATAGAGAAAAGTGTGGCAGAAAAGCG

Noncoding RNAs	
Terra	CCCTAACCCTAACCCTAACCCTAACCCTAACCCTAACCCTAACCCTAA
Terra-reverse	TTAGGGTTAGGGTTAGGGTTAGGGTTAGGGTTAGGGTTAGGGTTAGGG
Sine B2	GTCCTGAGTTCAATTCCCAGCAACCACATGGTGGCTCACAACCATC
Xist	CCAGCCATGTTTGCTCGTTTCCCGTGGATGTGCGGT

Supplementary Table S3. Characterize feature name, length, position and retrieved sequence of different transcripts for a single region of each rare high-peak signals which are presented in the sperm DRNA (blue star peak in Fig. 4) fraction as well in free-RNA fraction and the testes DRNA for sequence homology.

Region	samples	Gene ontology	library	p-value	Enriched genes	Number of total genes
3’	dRNA	positive regulation of flagellated sperm motility (GO:1902093)	GO Biological Process 2018	0.00002624	RNASE10;TACR2	27
		regulation of flagellated sperm motility (GO:1901317)		0.00006281	RNASE10;TACR2	
5’	dRNA	MP:0009833_absent_sperm_mitochondrial_sheath	MGI Mammalian Phenotype 2017	0.001399	Spata6	7
		MP:0009234_absent_sperm_head		0.002098		
		MP:0009834_abnormal_sperm_annulus_morphology		0.002448		
		MP:0008893_detached_sperm_flagellum		0.002797		
		MP:0002662_abnormal_cauda_epididymis_morphology		0.003146		
exon	dRNA	Spermatozoon	Jensen TISSUES	0.00004370	ODF1;AKAP4;AKAP3	101
		Spermatid		1.476e-11	SMCP;PRM1;ODF1;ODF2;TXNDC2; YBX2;HSPA2;PGK2; TNP2;AKAP4;AKAP3;H1FNT	
		Sperm_fibrous_sheath	Jensen COMPARTMENTS	0.000002472	PGK2;AKAP4;AKAP3	
		Sperm_flagellum		5.731e-7	ODF1;ODF2;PTCHD3;PGK2;AKAP4;AKAP3	
		Male_germ_cell_nucleus		0.001899	HSPA2;TNP2	
		MP:0004542_ impaired_acrosome_reaction	MGI Mammalian Phenotype 2017	5.671e-8	SMCP;ATP8B3;ODF1;TNP2;OAZ3	
		Male_infertilit	Jensen DISEASES	1.056e-10	CRISP2;PRM1;ODF1;TXNDC2; YBX2;HSPA2; PGK2;TNP2;AKAP4;AKAP3;OAZ3	
Promoter-TSS	dRNA	H3K36me3_testis_mm9	ENCODE Histone Modifications 2015	0.007206	SNORA62;ZC3H10;BC005537;9230105E05RIK; SNORD15B;PRM2;SCARNA10;RNF138;UBE2N; DBIL5;PHF7;1700017D01RIK	55
TTS	dRNA	Epididymitis	Jensen DISEASES	0.008968	TUBB4B	36
Intron	dRNA	MP:0011610_abnormal_primordial_germ_cell_apoptosis	MGI Mammalian Phenotype 2017	0.001724	BRIP1, WNT5A, ROR2	1147
		testis	Tissue Protein Expression from ProteomicsDB			

Supplementary Table S4. Gene set enrichment analysis of different regions using Enrichr tool in DRNA from sperm and testes.

Region	samples	Gene ontology	library	p-value	Number of total genes
5'	TD	dosage compensation by inactivation of X chromosome (GO:0009048)	GO Biological Process 2018	0.001753	921
exon	TD	RNA processing (GO:0006396)	GO Biological Process 2018	2.496e-8	7742
		protein ubiquitination (GO:0016567)		4.05E-09	
		Nucleoplasm	Jensen COMPARTMENTS	2.011e-17	
		RNA binding (GO:0003723)	GO Molecular Function 2018	1.565e-12	
		MP:0001925_male_infertility	MGI Mammalian Phenotype 2017	2.508e-7	
		centrosome (GO:0005813)	GO Cellular Component 2018	8.756e-16	
Promoter-TSS	TD	MP:0009834_abnormal_sperm_annulus_morphology	MGI Mammalian Phenotype 2017	0.002039	1852
Intron	TD	MP:0001925_male_infertility	MGI Mammalian Phenotype 2017	6.839e-12	8709
		MP:0006380_abnormal_spermatid_morphology		1.796e-9	
